# Acute 3,4-Methylenedioxymethamphetamine (MDMA) Toxicity Leading to Fulminant Hepatic Failure and Emergency Liver Transplantation

**DOI:** 10.7759/cureus.99716

**Published:** 2025-12-20

**Authors:** Maria E Batista, Miguel Barbosa, José Casimiro, César B Vieira, Nuno Germano

**Affiliations:** 1 Department of Critical Care Medicine, Hospital Curry Cabral, Unidade Local de Saúde de São José, Lisbon, PRT; 2 Department of Intensive Care, Hospital Curry Cabral, Unidade Local de Saúde de São José, Lisbon, PRT

**Keywords:** 4-methylenedioxymethamphetamine (mdma), amphetamine toxicity, drug-induced acute liver failure, hyperthermia, orthotopic liver transplantation

## Abstract

Recreational use of 3,4-methylenedioxymethamphetamine (MDMA, “ecstasy”) has increased across Europe, with rare but potentially fatal complications. We report a case of fulminant hepatic failure and multiorgan dysfunction following acute MDMA intoxication in a previously healthy 22-year-old man. One hour after ingestion, he developed seizures, hyperthermia (43 °C), and cardiovascular collapse. Laboratory results revealed severe metabolic acidosis, rhabdomyolysis (creatine kinase (CK) >360 000 U/L), acute kidney injury, disseminated intravascular coagulation, and rapidly progressive hepatic necrosis (aspartate aminotransferase (AST)/alanine aminotransferase (ALT) >3000 U/L, factor V <5%). Despite aggressive resuscitation, continuous renal replacement therapy, N-acetylcysteine infusion, plasma exchange, and hemoadsorption, hepatic failure progressed. On day 5, urgent orthotopic liver transplantation was performed. Postoperatively, immunosuppression and antimicrobial prophylaxis were initiated, and the patient gradually stabilised. This case highlights the potential for catastrophic systemic toxicity from MDMA, the importance of early recognition, intensive support, and the role of timely liver transplantation in ensuring survival.

## Introduction

3,4-Methylenedioxymethamphetamine (MDMA), or ecstasy, use as a recreational drug has been increasing in Europe in the last decades [1]. Aside from its intended psychological effects, the use of ecstasy can be followed by symptoms of intoxication; complications include toxic hepatic damage up to acute hepatic failure [1,2].

The extent of ecstasy-associated severe hepatic damage remains unknown, and the exact pathophysiologic mechanisms are not fully understood [2,3]. MDMA is primarily metabolized by hepatic cytochromes, but some metabolic intermediates remain pharmacologically active and may exert greater hepatotoxic effects than the parent compound itself [4].

We describe a case of life-threatening multiorgan failure following acute MDMA ingestion, detailing the sequence of systemic deterioration and complex critical care management leading to liver transplantation.

## Case presentation

History of presenting illness

A previously healthy 22-year-old man was brought to the emergency department after reportedly ingesting MDMA during a social event. Within an hour, he developed generalised tonic-clonic seizures followed by profuse sweating and agitation, and collapsed. On arrival, emergency medical staff recorded a core temperature of 43°C, prompting immediate initiation of external cooling measures.

On admission to the resuscitation unit, the patient was unconscious (GCS 6), tachycardic (150 bpm), tachypnoeic (30 breaths/min), and hypotensive (mean arterial pressure 62 mmHg despite intravenous fluids). He was intubated and mechanically ventilated for airway protection and respiratory failure. Pupils were mid-dilated and sluggishly reactive. Marked muscle rigidity and shivering were observed. He was promptly transferred to the ICU.

Investigation

Initial laboratory workup demonstrated a severe mixed metabolic and respiratory acidosis (pH 7.12, pCO₂ 59 mmHg, lactate 7.5 mmol/L); serum creatinine was 2.04 mg/dL and potassium 5.0 mmol/L, indicating early renal impairment. Cardiac biomarkers showed troponin-T 1000 ng/L and the ECG showed no ischaemic changes. The most striking abnormality during the first 12 hours was a rapidly progressive rise in creatine kinase (CK) and myoglobin, consistent with massive rhabdomyolysis. CK values exceeded 360 000 U/L within 72 hours, accompanied by myoglobinuria and progressive oliguria, evolving to anuric acute kidney injury (AKIN stage 3). Serum lactate rose steadily to 13.5 mmol/L despite adequate oxygenation, reflecting systemic mitochondrial dysfunction and severe shock.

Liver function tests showed a dramatic cytolytic pattern: aspartate transaminase (AST) and alanine transaminase (ALT) increased from 25/18 U/L on admission to >3000 U/L within 72 hours, while total bilirubin rose to 7.84 mg/dL and ammonia reached 140 µmol/L (Table 1). Coagulation studies revealed profound derangement, with INR 4.1, aPTT 121 seconds, fibrinogen 0.9 g/L, and factor V <5%, fulfilling criteria for acute fulminant hepatic failure.

**Table 1:** Evolution of biochemical parameters over time. AST: Aspartate aminotransferase; ALT: Alanine aminotransferase; LDH: Lactate dehydrogenase; CK: Creatine kinase; Tbil: Total bilirubin; Dbil: Direct bilirubin.

Parameter (units; normal range)	Day 0	Day 1	Day 2	Day 3	Day 4	Day 5
AST (U/L; normal range: 5.0-34.0)	25	800	1309	3623	1530	1544
ALT (U/L; normal range: 0.0-58.0)	18	152	1097	3310	1273	741
LDH (U/L; normal range: 125-220)	376	2981	2213	4252	1941	1475
CK (U/L; normal range: 30-200)	40161	56830	29651	363296	53680	40500
Tbil (mg/dL; normal range: 0.20-1.20)	0.72	1.09	2.2	4.02	5.84	7.84
Dbil (mg/dL; normal range: 0.00-0.50)	-	-	-	1.62	2.65	4.68

Serial arterial blood gases showed persistent metabolic acidosis despite mechanical ventilation, and serum lactate dehydrogenase (LDH) increased to >4000 U/L, supporting a diagnosis of tissue necrosis and multiorgan failure. Complete blood count revealed progressive leukocytosis (25 × 10⁹/L) and thrombocytopenia (nadir 22 × 10⁹/L).

**Figure 1:**
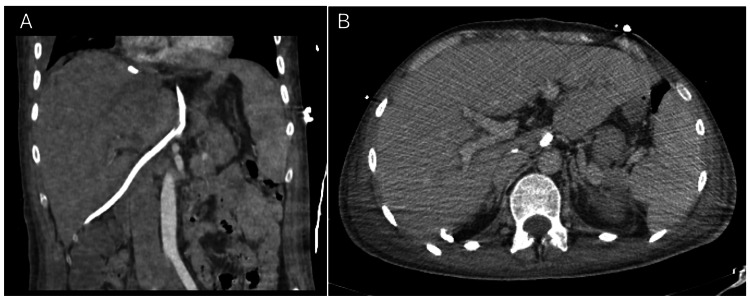
Contrast-enhanced CT scan of the upper abdomen. Contrast-enhanced CT scan of the upper abdomen in the arterial phase (A) and portal venous phase (B). The hepatic parenchyma appears heterogeneous, with areas of decreased attenuation compatible with hepatocellular necrosis (A). There is mild periportal oedema, with no evidence of a focal mass or biliary dilatation (B). The hepatic vasculature is patent. No ascites or focal collections are seen.

Imaging played a supportive role: cranial and cervical CT scans were normal, excluding intracranial pathology as a cause of coma; chest X-rays later revealed bilateral pulmonary infiltrates and right-sided pleural effusion, probably related to fluid resuscitation. Abdominal ultrasound and Doppler demonstrated hepatomegaly with diffuse hypoechogenicity and preserved flow, excluding obstructive vascular disease; CT imaging confirmed diffuse hepatic hypodensity with patent hepatic vasculature (Figure 1).

Microbiological cultures were repeatedly negative, suggesting that the systemic inflammatory response and coagulopathy were primarily due to toxic injury resulting in ischaemia rather than infectious mechanisms.

Comprehensive forensic toxicology confirmed the presence of multiple amphetamine derivatives in both serum and whole blood samples. Quantitative analysis revealed high concentrations of MDMA (up to 308 ng/mL), along with MDA (7.8 ng/mL), methamphetamine (1378 ng/mL), and amphetamine (24 ng/mL) at first sampling. Serial measurements over three consecutive days demonstrated a rapid decline in serum concentrations, consistent with acute ingestion followed by metabolic clearance. The detection of both MDMA and its metabolite MDA supports recent, substantial exposure, correlating temporally with the onset of hyperthermia and multiorgan failure. No other drugs or alcohol were detected, reinforcing MDMA as the primary agent responsible for the observed toxicity.

Overall, the integration of laboratory and imaging findings confirmed fulminant hepatic failure secondary to MDMA toxicity, complicated by rhabdomyolysis, acute kidney injury, disseminated intravascular coagulation and refractory shock.

Differential diagnosis

At presentation, the combination of extreme hyperthermia (43°C), generalised muscle rigidity, seizures, and rapid cardiovascular collapse prompted consideration of several hypermetabolic syndromes, including malignant hyperthermia, neuroleptic malignant syndrome (NMS), and serotonin syndrome, in addition to toxic or viral hepatitis as potential causes of hepatic failure.

Malignant hyperthermia was initially suspected due to the extreme hyperthermia (43°C) and muscle rigidity, but was deemed less likely as there was no exposure to triggering anaesthetic agents or depolarising neuromuscular blockers and no family history of the condition.

NMS was also considered based on the clinical presentation. However, the patient had no history of antipsychotic or dopamine antagonist exposure, and the onset was too rapid, which typically evolves over 1-3 days. In addition, clonus and hyperreflexia, hallmarks of serotonergic excess, were present, whereas NMS more often produces lead-pipe rigidity with hyporeflexia.

The constellation of hyperthermia, neuromuscular hyperactivity (rigidity, clonus, tremor), autonomic instability, and altered mental status strongly supported a diagnosis of serotonin syndrome, likely precipitated by massive MDMA ingestion. This diagnosis also aligned with the pathophysiological mechanism of MDMA, which acts as a potent serotonin-releasing agent and monoamine reuptake inhibitor, leading to excess synaptic serotonin and downstream hypermetabolic effects.

Acute viral or autoimmune hepatitis was ruled out by negative serology and absence of autoantibodies, leaving MDMA-induced hepatotoxicity as the unifying diagnosis.

Taken together, the multisystem involvement, neurological, hepatic, renal, and haematological, and the rapid sequence of deterioration following MDMA ingestion were most consistent with an acute MDMA-induced serotonin syndrome presenting as a hypermetabolic crisis, culminating in fulminant hepatic failure and multiorgan dysfunction.

Treatment

**Figure 2:**
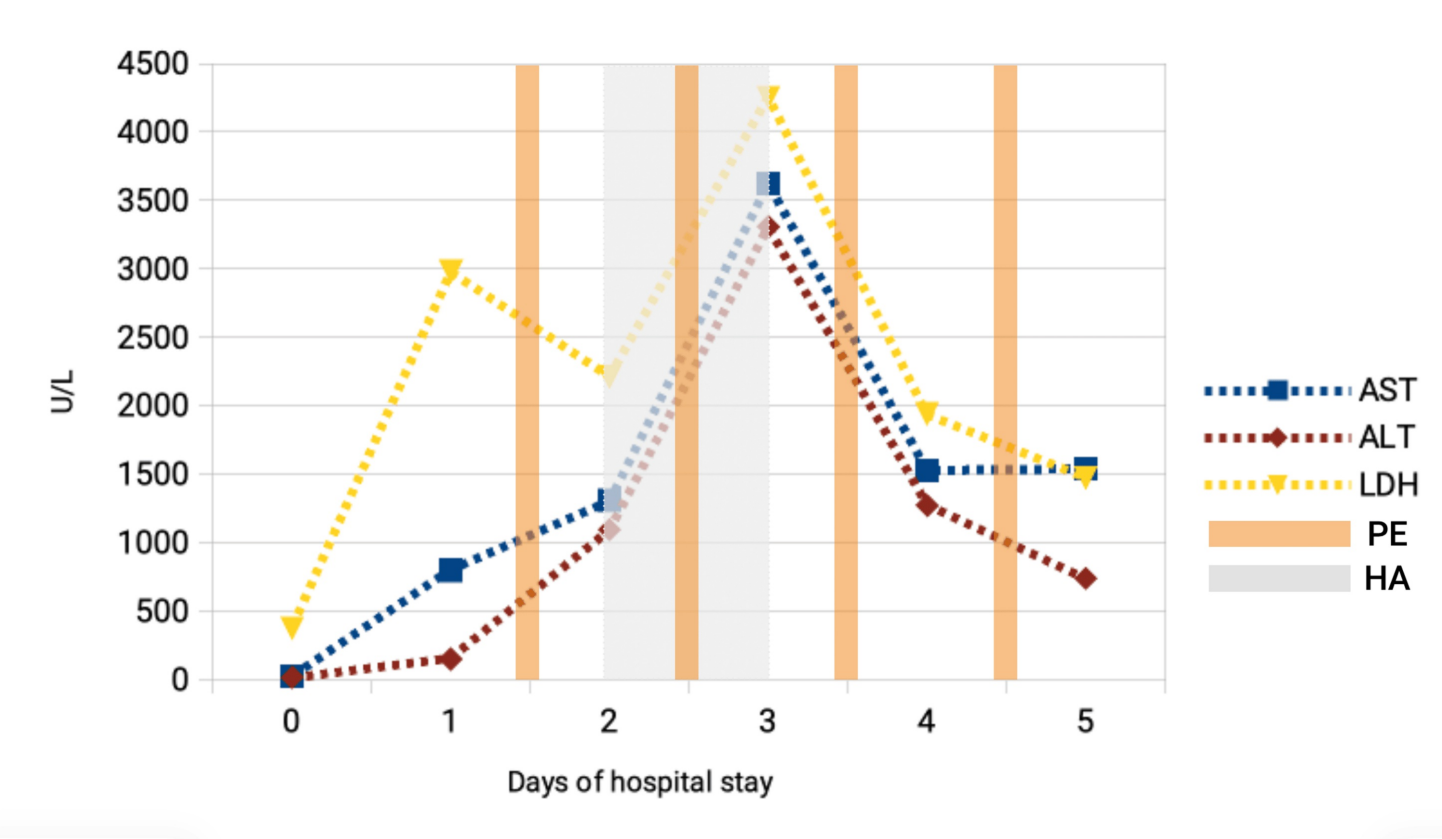
Trend of aspartate aminotransferase (AST), alanine aminotransferase (ALT), and lactate dehydrogenase (LDH) during the hospital stay. PE: Plasma exchange; HA: Hemoadsorption.

The management of MDMA-induced acute liver failure is primarily supportive. In our case, initial measures focused on hemodynamic stabilization, rapid control of hyperthermia through external cooling, and correction of metabolic acidosis through early continuous renal replacement therapy. Early administration of intravenous N-acetylcysteine was initiated, given its potential antioxidant and hepatoprotective effects in drug-induced liver injury, despite limited evidence specific to MDMA [5,6]. Continuous renal replacement therapy was also employed to manage severe rhabdomyolysis and acute kidney injury. When hepatic function continued to deteriorate, therapeutic plasma exchange was introduced as a bridge to transplantation, aiming to remove circulating cytokines, bilirubin, and toxins and to correct coagulopathy. A total of four plasma exchange sessions were performed, each replacing 1.5-2 plasma volumes with fresh frozen plasma. In parallel, a hemoadsorption cartridge (CytoSorb®) was incorporated into the renal replacement circuit for additional cytokine clearance. Figure 2 illustrates the longitudinal trends of AST, ALT, and LDH over the hospital course, alongside the timing of key supportive interventions given as a bridge to transplant. Coagulopathy and hemorrhagic complications required massive transfusion and correction with fibrinogen, cryoprecipitate, and platelets. On the fifth day of hospitalization, due to worsening hepatic encephalopathy and a precipitous decline in synthetic function (factor V <20%, INR >4), the patient underwent urgent orthotopic liver transplantation. Postoperatively, immunosuppression with tacrolimus and corticosteroids was introduced, along with prophylaxis using hepatitis B immunoglobulin and broad-spectrum antimicrobial coverage. The multidisciplinary management approach was essential for patient survival, consistent with the recommendations from previously reported severe MDMA hepatotoxicity cases [1,7,8].

Outcome and follow-up

The transplanted liver function progressively improved, with factor V >80% and bilirubin gradually decreasing. Renal function recovered. The patient was discharged from intensive care several weeks later with stable graft function. At follow-up three months later, he had returned to daily activities with ongoing hepatology and transplant clinic monitoring.

## Discussion

MDMA-induced hepatotoxicity exhibits a wide clinical spectrum, ranging from mild, transient enzyme elevation to fulminant hepatic failure. In the retrospective review by Andreu V et al. (1998), ecstasy accounted for a small but significant proportion of acute liver failure cases in young adults; however, all patients recovered without transplantation, highlighting the usually self-limited nature of this toxicity [2]. Similarly, Aknine X et al. (2004) described a case of ecstasy-induced toxic hepatitis that resolved completely after cessation of drug use [3]. More recent reports, such as Sharma NR et al. (2022) and Jha A et al. (2022), also document reversible hepatic injury managed conservatively [5,9]. In contrast, Atayan Y et al. (2015) and Politi C et al. (2021) presented rare but fatal or transplant-requiring cases, illustrating that fulminant failure, although exceptional, can occur [1,7].

Our patient’s rapid progression to multiorgan failure, with hyperthermia exceeding 43 °C, disseminated coagulopathy, and massive rhabdomyolysis, represents the extreme end of this clinical spectrum. Unlike most previously published cases, he required urgent orthotopic liver transplantation after extracorporeal bridging therapies (plasma exchange, cytokine adsorption). His recovery contrasts with the fatal outcomes described in the Politi C et al. (2021) series [7], demonstrating that timely recognition and early referral to a transplant center can be life-saving. Mechanistically, these severe forms may reflect combined hepatocellular hypoxia from hyperthermia, oxidative stress due to reactive MDMA metabolites, and mitochondrial dysfunction, as described by Carvalho M et al. (2010) and Capela JP et al. (2022) [6,10]. This case therefore contributes to the limited body of evidence showing that, while MDMA hepatotoxicity is rare, it can progress rapidly to life-threatening liver failure requiring transplantation. However, the optimal timing for transitioning from conservative management to liver transplantation remains unclear.

## Conclusions

This case illustrates the potential for catastrophic systemic toxicity following acute MDMA ingestion, even in young, previously healthy individuals. Although most reported cases of MDMA-induced hepatotoxicity resolve spontaneously, a minority progress to fulminant hepatic failure and multiorgan dysfunction requiring emergency liver transplantation. Early recognition is essential to guide timely intervention. Supportive measures, including aggressive cooling, N-acetylcysteine, plasma exchange, and hemoadsorption, may provide a bridge to transplantation, but definitive survival depends on prompt referral to a transplant centre. This rare case reinforces the need for heightened clinical awareness of MDMA toxicity and public education regarding its potentially fatal consequences.

## References

[1] Atayan Y, Çağın YF, Erdoğan MA, Harputluoglu MM, Bilgic Y (2015). Ecstasy induced acute hepatic failure. Case reports. Acta Gastroenterol Belg.

[2] Andreu V, Mas A, Bruguera M (1998). Ecstasy: a common cause of severe acute hepatotoxicity. J Hepatol.

[3] Aknine X (2004). Ecstasy-induced toxic hepatitis. Presse Med.

[4] Cajanding RJ (2019). MDMA-associated liver toxicity: pathophysiology, management, and current state of knowledge. AACN Adv Crit Care.

[5] Sharma NR, Sharma B, Lamichhane S, Pokhrel M, Gautam S (2022). A case of ecstasy-induced acute hepatic injury. Cureus.

[6] Carvalho M, Pontes H, Remião F, Bastos ML, Carvalho F (2010). Mechanisms underlying the hepatotoxic effects of ecstasy. Curr Pharm Biotechnol.

[7] Politi C, Gabbin A, Cecchetto G, Montisci M, Viel G, Pascali P (2021). A case study on MDMA. Two fatal cases involving young adults. Aust J Forensic Sci.

[8] Makunts T, Abagyan R (2024). Hepatic injury and hepatic failure adverse events in 3,4-methylenedioxymethamphetamine users reported to the FDA Adverse Event Reporting System. Front Psychiatry.

[9] Jha A, Patel M, Sahu A, Shahm A, Bohra S (2022). Acute liver failure - an interesting case report. J Clin Exp Hepatol.

[10] Capela JP, Carvalho FD (2022). A review on the mitochondrial toxicity of "ecstasy" (3,4-methylenedioxymethamphetamine, MDMA). Curr Res Toxicol.

